# Meat and Seafood Consumption in Relation to Plasma Metabolic Profiles in a Chinese Population: A Combined Untargeted and Targeted Metabolomics Study

**DOI:** 10.3390/nu9070683

**Published:** 2017-06-30

**Authors:** Yonghai Lu, Li Zou, Jin Su, E. Shyong Tai, Clare Whitton, Rob M. van Dam, Choon Nam Ong

**Affiliations:** 1 Saw Swee Hock School of Public Health, National University of Singapore, Singapore 117549, Singapore; ephluyng@nus.edu.sg (Y.L.); ephzouli@nus.edu.sg (L.Z.); sujin@nus.edu.sg (J.S.); clarewhitton@nus.edu.sg (C.W.); 2 Institute of Nutrition and Health, Qingdao University, Qingdao 266021, Shandong, China; 3 Division of Endocrinology, National University Hospital, Singapore 119074, Singapore; e_shyong_tai@nuhs.edu.sg; 4 Department of Medicine, Yong Loo Lin School of Medicine, National University Health System, Singapore 119228, Singapore; 5 NUS Environmental Research Institute, National University of Singapore, Singapore 117411, Singapore

**Keywords:** metabolomics, amino acids, fatty acids, CMPF, glucose metabolism, dietary pattern, red meat, poultry, fish, shellfish

## Abstract

We examined the relationship between different patterns of meat and seafood consumption and plasma metabolic profiles in an Asian population. We selected 270 ethnic Chinese men and women from the Singapore Prospective Study Program based on their dietary habits assessed with a validated food frequency questionnaire. Participants were divided into four subgroups: high meat and high seafood (*n* = 60), high meat and low seafood (*n* = 64), low meat and high seafood (*n* = 60), and low meat and low seafood (*n* = 86) consumers. Plasma metabolites were measured using both targeted and untargeted mass spectroscopy-based analyses. A total of 42 metabolites differed significantly by dietary group. Higher concentrations of essential amino acids, polyunsaturated fatty acids, and d-glucose were found in high meat and/or seafood consumers as compared with the group with a low consumption of these animal foods. Red meat, poultry, fish, shellfish, soy products, and dairy were each correlated with at least one differential metabolite (*r* = −0.308 to 0.448). Some observations, such as the correlation between fish and 3-carboxy-4-methyl-5-propyl-2-furanpropanoic acid (CMPF), confirmed previous studies. Other observations, such as the correlation between shellfish and phosphatidylethanolamine (p36:4), were novel. We also observed significant correlations between plasma metabolites and clinical characteristics, such as CMPF with fasting blood glucose (*r* = 0.401). These findings demonstrate a significant influence of meat and seafood consumption on metabolic profiles in the Asian population.

## 1. Introduction

Changes in dietary consumption and energy expenditure that characterize the “nutrition transition” have been considered as the causal factors underlying non-communicable diseases in developing countries [1]. Economic growth as a major driver of nutrition transition has a significant effect on the consumption of different food aggregates [2]. In Asia, the effect of economic factors on the nutrition transition has been particularly apparent [3,4]. For example, China is experiencing an even more rapid shift in diet, with increased consumption of animal products [5]. Officially, the Chinese average annual meat consumption increased from 19.33 kg in 1999 to 23.96 kg in 2008 (National Bureau of Statistics 2000–2009) [2]. Concurrent with this transition, obesity is increasing in China, as well as widespread diet-related chronic diseases including cardiovascular diseases and diabetes.

The interactive links between diet and the risk of chronic diseases have been the focus of international research efforts over the last few decades. For example, the intake of meat, particularly red meat, has been associated with an increased risk of type 2 diabetes [6], coronary heart disease [7], and certain forms of cancer [8] in epidemiologic studies. In contrast, the intake of fish appears to be protective against these diseases, especially on cardiovascular disease [9,10], whereas it shows both inverse [11,12] and direct [13,14] associations with the risk of type 2 diabetes. Most of these studies have used dietary questionnaires to assess meat and fish intake, but the accuracy of food intake data can be influenced by systematic and random errors due to misreporting. The use of biomarkers may improve the accuracy of dietary intake assessment and help to classify subjects according to the type of food and amount consumed. To date, a series of dietary biomarkers have been identified for red meat [15], salmon, broccoli [16], cruciferous vegetables [17], citrus fruit [18], and coffee and tea [19].

Different diet compositions may have different effects on disease risk. By taking into account the interactions between nutrients, nutritional studies are increasingly shifting their focus from studying single nutrients or foods to the exploration of the whole dietary pattern. In 2015, Schmidt et al. compared the plasma metabolic profiles of 379 male meat eaters, fish eaters, vegetarians, and vegans from the European Prospective Investigation into Cancer and Nutrition-Oxford (EPIC-Oxford) cohort [20]. They found that meat eaters had the highest concentrations of glycerophospholipids and sphingolipids compared with fish eaters and vegetarians. In 2017, Cheung et al. identified three acylcarnitines (acetylcarnitine, propionylcarnitine, and 2-methylbutyrylcarnitine) as generic indicators of meat and fish intake in the EPIC study [21]. In Asia, only one study has been published on the metabolic profiles in Chinese people with different habitual diets, e.g., meat eaters and vegetarians [22]. This study found clear associations between dietary patterns and metabolic profiles and elevated hippurate and reduced taurine and methylhistidine were identified as specific biomarkers for lactovegetarians.

In the present study, we examined patterns of meat and seafood consumption in relation to plasma metabolic profiles in a Chinese population by using both untargeted and targeted metabolomics strategies. The results can contribute to the identification of novel dietary biomarkers and potential mediators of the associations between diet and health conditions. In the targeted metabolomics, we focused on amino acid and fatty acid profiles, as meat and fish are important sources of protein rich in essential amino acids and polyunsaturated fatty acids [23,24].

## 2. Materials and Methods

### 2.1. Study Population and Diet Groups

**Figure 1:**
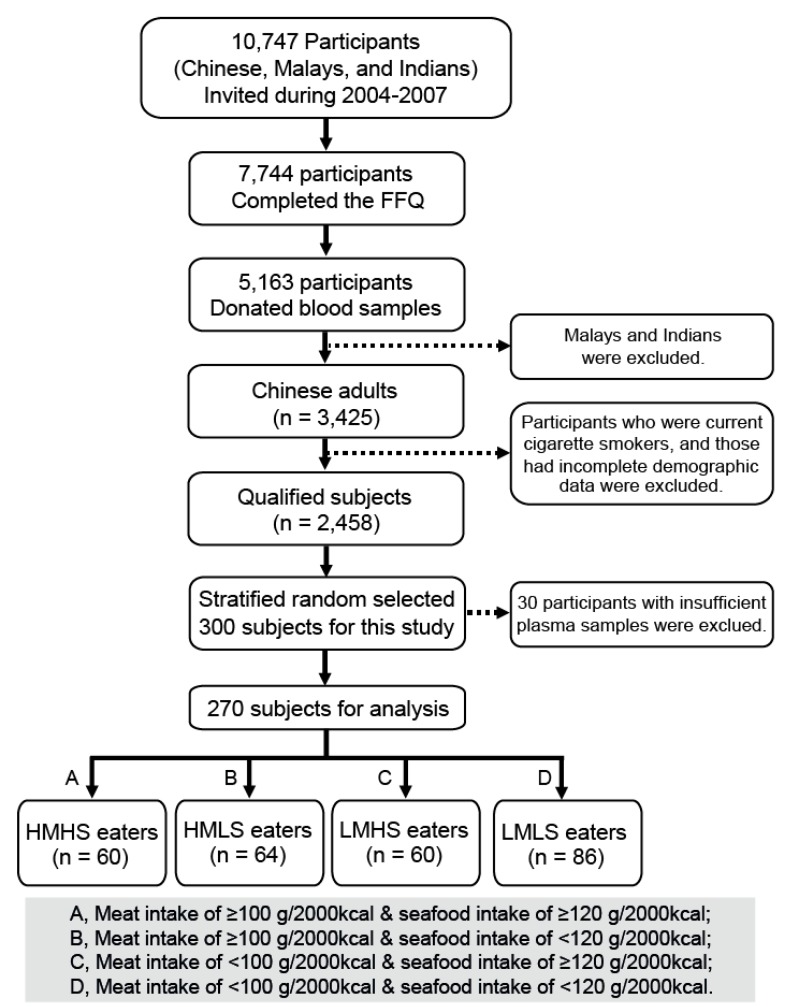
Flow diagram of the study participant selection from the Singapore Prospective Study Program cohort. HMHS, high meat and high seafood; LMLS, low meat and low seafood; HMLS, high meat and low seafood; LMHS, low meat and high seafood.

Participants investigated in this study were from the Singapore Prospective Study Program (SP2), which is a population-based cross-sectional study in a multiethnic population in Singapore. The detailed population selection and methodology for SP2 have been published previously [25,26,27]. Briefly, 10,747 volunteers including Chinese, Malays, and Indians were invited from 2004 to 2007. Among them, 7744 participants completed the food frequency questionnaires (FFQ), of which 5163 donated blood samples. The FFQ used in this study was slightly adapted from the FFQ used by the Health Promotion Board (HPB) in the Report of the National Nutrition Survey 2004, Singapore [28]. Only ethnic Chinese were selected for this study, as they make up over 70% of the resident Singapore population (http://www.singstat.gov.sg/) and most do not have strong dietary restrictions due to their religion or cultural beliefs. We also excluded participants who were current cigarette smokers to avoid residual confounding by tobacco dose, and those who had incomplete demographic data. Because of logistic and funding constraints, we randomly selected 300 participants within strata of high meat consumption, high fish consumption, and low meat and fish consumption from the final qualified population (*n* = 2458) for the current analysis. Among them, 30 participants had insufficient plasma samples for analysis, resulting in a total number of 270 participants. Based on the objective of the current study (i.e., to compare the metabolic profiles in Chinese with different patterns of meat and seafood consumption), the participants were divided into four groups: high meat and high seafood (HMHS, *n* = 60), high meat and low seafood (HMLS, *n* = 64), low meat and high seafood (LMHS, *n* = 60), and low meat and low seafood (LMLS, *n* = 86) consumers. A flow diagram of study participant selection is shown in Figure 1. The cut-off values of meat (100 g/2000 kcal per day) and seafood (120 g/2000 kcal per day) intake reflect consumption of at least one serving per day in Singapore residents. Here, meat consumption was defined as the summed intake of red meat and poultry, and seafood consumption was defined as the summed intake of fish and shellfish. The food intakes in our study were adjusted to an average value of 2000 kcal per day in order to normalise dietary exposures across this population, and we also found that the converted food intakes by 2000 kcal per day showed a closer association with the plasma metabolic profiles than the original estimated food intakes (grams per day) (Supplementary Figure S1). Informed consent was obtained from all participants. Ethics approval was obtained from the Institutional Review Boards at the National University of Singapore (NUS-IRB 12-282). The study was conducted in accordance with the Helsinki Declaration of 1975, as revised in 1983.

### 2.2. Dietary Assessment

Dietary information of the participants was estimated by an interviewer-administered semi-quantitative 159-item FFQ, previously validated against three 24-h dietary recalls for energy and selected macronutrients with correlation coefficients ranging from 0.39 to 0.58 [29]. Briefly, for each food item, the participant was asked to report intake over the past one month as frequency either per day, per week, per month, or rarely/never. Pre-defined standard portion sizes were included and expressed in terms of cups, spoons, and slices, and corresponding measuring utensils were supplied to aid estimation. Derived variables on estimated amount of ingredients in each FFQ item were used. Energy values (kcal) for each FFQ item were provided by the Singapore Health Promotion Board. For each participant, the FFQ responses were converted to grams per 2000 kcal by multiplying the daily frequency based on portion size (grams), then dividing by the daily energy intake (kcal)/2000 (which approximately is the average energy intake of the study population).

### 2.3. Plasma Collection and Pretreatment

The morning fasting antecubital venous blood was taken at the participants’ homes. After collection, blood samples were immediately placed on ice during transport to the laboratory and processed within 4 h to obtain plasma samples by centrifugation at 2500× *g* for 15 min at 4 °C. Plasma samples were stored at −80 °C until analysis. For untargeted analysis, plasma samples were allowed to thaw at 4 °C, and were then processed as described previously [30,31]. Briefly, 50 μL of plasma was diluted with 750 μL of ice-cold methanol containing 30 μg/mL *N*-(9-Fluorenylmethoxycarbonyl)-glycine as an internal standard. After centrifugation at 20,817× *g* for 15 min at 4 °C, the supernatant fraction was filtered by Thermo Scientific™ national 750 µL micro-centrifugal filters (PTFE membrane, 0.2 µm pore size, non-sterile). The filtrate was collected and aliquoted into two portions: 100 μL and 20 μL, and dried by 99.9% purity of nitrogen. The dried samples obtained from 100 μL portions were dissolved in 200 μL of acetonitrile/water (95:5, *v*/*v*) for liquid chromatography-mass spectrometry (LC-MS) analysis. The dried samples obtained from 20 μL portions were derivatized with 100 μL methoxyamine (2 mg/mL) in pyridine for 2 h at 40 °C and then with 150 μL *N*-methyl-*N*-trimethyl-silyl-trifluoroacetamide (40 °C × 16 h) for gas chromatography-mass spectrometry (GC-MS) analysis. The pooled quality control (QC) samples were prepared by mixing equal amounts (10 μL) of each plasma sample.

### 2.4. Untargeted Analysis

Untargeted analysis was performed on both LC-MS and GC-MS as our previously described protocol [32,33], with slight modifications. LC-MS analysis was performed on an Agilent 1290 ultrahigh pressure LC system (Agilent Technologies, Waldbronn, Germany) coupled to a 6540 Q-ToF mass detector (Agilent Technologies, Santa Clara, CA, USA) equipped with an electrospray ionization source, in both the positive and negative ion modes. The sample (2 μL) was separated on an Agilent rapid resolution HT Zorbax SB-C18 column (2.1 × 50 mm, 1.8 µm) at 50 °C. Mobile phases A (water with 0.1% formic acid) and B (methanol/acetonitrile/water (9:4:1, *v*/*v*/*v*) with 0.1% formic acid) were employed in the positive ion mode, whereas 0.1% formic acid was replaced by 10 mmol/L ammonium formate in the negative ion mode. The gradient program was: 0–1 min, 5% B; 1–6 min, 5–80% B; 6–9 min, 80–90% B; 9–13 min, 90–100% B; 13–16 min, 100% B; 16–18 min, 100–5% B. Mass data were collected between *m*/*z* 100 and 1000 at a rate of two scans per second. The ion spray voltage was set at 4000 V, and the heated capillary temperature was maintained at 325 °C. The drying gas and nebulizer nitrogen gas flow rates were 10.0 L/min and 40 psi, respectively. MS/MS analysis was carried out to study the structure of potential biomarkers. GC-MS analysis was performed on an Agilent 7683B Series Injector (Agilent Technologies, Santa Clara, CA, USA) coupled to an Agilent 7890A Series GC system (Agilent Technologies, Santa Clara, CA, USA) and a 7200 Q-TOF mass detector (Agilent Technologies, Santa Clara, CA, USA). A fused-silica capillary column HP-5MSI (30 m × 0.25 mm i.d., 0.25 μm film thickness) was used for sample separation. The injector was kept at 250 °C. One μL of the sample was injected at the pulsed split ratio of 20:1 for individual analysis. Helium was used as the carrier gas with a constant flow rate of 1 mL/min. The GC oven temperature was maintained at 50 °C for 1 min, and then increased to 250 °C at a rate of 8 °C/min and further increased at 25 °C/min to 300 °C and was held for 5 min. The transfer line temperature was kept at 280 °C. Detection was achieved using MS in electron impact mode (70 eV) and full scan monitoring (*m*/*z* 50 to 550).

The 270 samples were randomized across platform run days. The stability of LC-MS and GC-MS during running was examined and evaluated by using the pooled QC samples [34] (Supplementary Figure S2), which were analyzed at the beginning, the end, and randomly throughout the whole assay. The variations of mass accuracy (<10 mDa), retention time (<0.3 min), and peak area (relative standard deviation <20%) for a subset of peaks covering a range of masses, retention times, and intensities across the QC samples demonstrated that our analytical methods provided measurement stability and reliability for the present study (Supplementary Table S1). Structure identification of metabolic candidates followed our previously described protocol [31,32]. The structures of metabolites were confirmed using commercial standards, such as d-glucose and 3-carboxy-4-methyl-5-propyl-2-furanpropanoic acid (CMPF).

### 2.5. Targeted Analysis

The methods for targeted analysis of the amino acids and total fatty acids in plasma were previously described [35,36], and are described in the Supplementary Materials and Methods.

### 2.6. Statistical Analysis

Mass spectral data of the metabolite profiling analysis were exported as mzData files by Agilent MassHunter Qualitative analysis B.06.00, and pretreated by open-source software MZmine 2 for peak detection, peak alignment, and data normalization using an internal standard. After screening of the metabolic features using the “80% rule” and replacement of the missing values (i.e., zeros) by 1/2 minimum [37,38], the preprocessed data were diagnosed in histogram plots to be skewed and were therefore transformed into log2-scale to meet the assumption of normality before the subsequent statistical analysis. The differences in the levels of metabolites between diet groups were examined using both one-way ANOVA and Student’s *t*-test. Participant and blood sample characteristics were compared among diet groups by one-way ANOVA for continuous variables, and by the chi-square test for categorical variables. The false discovery rate method of Benjamini and Yekutieli was used to correct for multiple hypothesis testing and reduce false positives [39]. The associations among dietary intakes, blood parameters and plasma metabolites were examined by Pearson correlation analysis with Bonferroni correction. Receiver operating characteristic (ROC) analysis was applied to evaluate the predictive potential of plasma metabolites for dietary patterns. *p*-values < 0.05 were considered significant.

## 3. Results

### 3.1. Participant and Blood Sample Characteristics

A selection of 270 participants from the SP2 cohort was investigated (Figure 1), and they were divided into four dietary groups: HMHS, HMLS, LMHS, and LMLS consumers (Table 1). There were no age, sex, and BMI differences between participants in the four groups. With regard to the clinical biochemical assays, HMHS and LMHS consumers had higher fasting blood glucose levels than LMLS consumers. Data on consumption of the 12 major food groups (including red meat, poultry, fish, shellfish, vegetables, fruits, whole grains, refined grains, soy products, dairy, eggs, nuts, and seeds) in typical Singapore Chinese diet are shown in Supplementary Table S2. Low meat consumers (including LMHS and LMLS) tended to have higher intakes of fruits, whole grains, and soy products than high meat consumers (including HMHS and HMLS). There was no significant difference in the total energy intake among the four dietary groups.

### 3.2. Differences of Plasma Metabolites among Four Dietary Groups

Using untargeted analysis, we identified 33 differential metabolites among the four dietary groups (the corrected *p-*value < 0.05), including d-glucose, CMPF, and 31 glycerophospholipids (Table 2 and Supplementary Figure S3). HMHS, HMLS, and LMHS consumers had higher levels of arachidonic acid (AA), eicosapentaenoic acid (EPA), and docosahexaenoic acid (DHA)-content glycerophospholipids, as well as G-glucose and CMPF than LMLS consumers, while they had lower levels in non-AA/EPA/DHA-content glycerophospholipids compared to LMLS consumers. With the targeted analysis of amino acids (*n* = 16) (Supplementary Table S3) and total fatty acids (*n* = 19) (Supplementary Table S4), six amino acids (glycine, hydroxyproline, lysine, threonine, tryptophan, and valine) and three total fatty acids (AA, EPA, and DHA) showed significantly different levels among the four dietary groups (Table 2 and Supplementary Figure S3). The data indicated that AA, EPA, DHA, lysine, threonine, tryptophan, valine, and hydroxyproline were higher in HMHS, HMLS, and LMHS consumers compared to LMLS consumers, whereas glycine was lower in HMHS, HMLS, and LMHS consumers.

**Figure 2:**
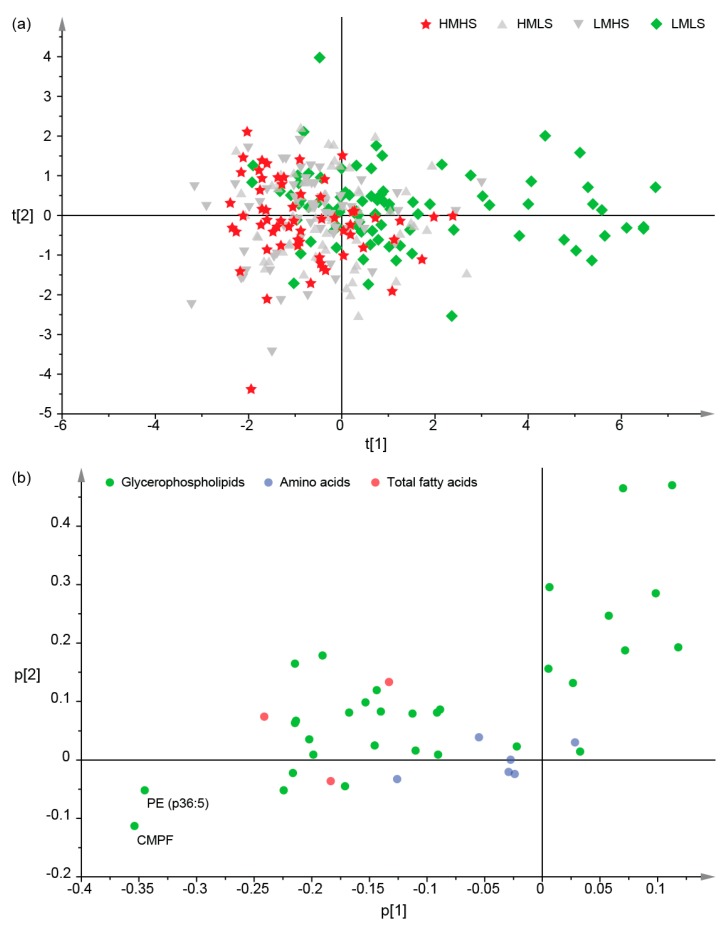
Principal component analysis of 42 differential metabolites. (**a**) Score scatter plot for principal components 1 and 2 by diet groups. Each point represents a participant. R2X (cum) = 0.768, Q2 (cum) = 0.425; (**b**) Loading plot for principal components 1 and 2. Each point represents a metabolite.

Principal component analysis (PCA) was further performed to develop a visual plot for the evaluation of the resemblance and difference in plasma metabolites among the four diet groups on the basis of the above-mentioned 42 differential metabolites. Seven principal components were retained for further inspection, and together they explained 76.8% of the total variation. The score plot of principal component 1 against 2 showed the best separation between the diet groups (Figure 2a), in which LMLS consumers were generally separated from the other three diet groups, whereas HMHS, HMLS, and LMHS consumers were still mixed together. The separation was mainly seen on principal component 1, which explained 35.7% of the total variation. The loading plot of the principal components 1 and 2 showed that the separation of LMLS consumers from the other groups was due to the lower concentrations of phosphatidylethanolamine (PE) (p36:5) and CMPF (Figure 2b).

**Figure 3:**
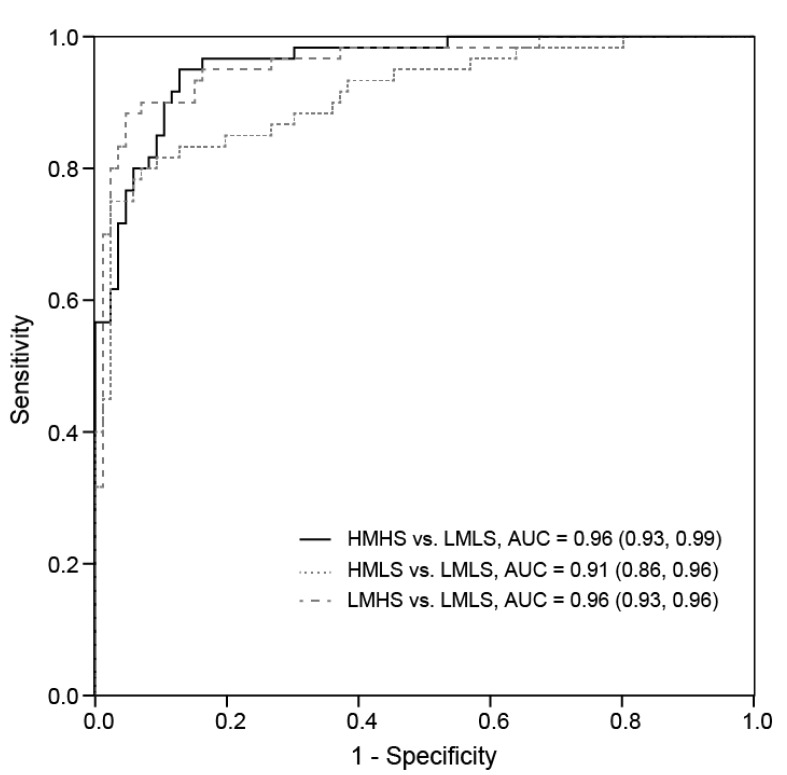
Receiver operating characteristic (ROC) curve of the combination of 42 differential metabolites by diet groups.

ROC curves were produced by using binary logistic regression to determine whether a panel of the above 42 differential metabolites could discriminate between high and low meat and/or seafood consumers (Figure 3). The area under the curve (AUC) (95% CI) values of 0.96 (0.93, 0.99), 0.91 (0.86, 0.96), and 0.96 (0.93, 0.99) were achieved by comparing LMLS consumers with HMHS, HMLS, and LMHS consumers, respectively, which represented excellent ROC curves according to the accepted classification of biomarker utility. However, we found that the assessment of each metabolite separately had much lower AUC values, ranging from 0.51 to 0.79 (Table 2). To enlarge the potential link of the individual metabolite with the consumption of meat and seafood, we further examined the AUC values of 42 metabolites by comparing the top (1st, *n* = 54) and the bottom (5th, *n* = 54) quintiles of total meat and seafood consumption (Supplementary Table S5). AUC values significantly increased in new models, and 12 metabolites had AUC values of >0.8, including EPA, DHA, phosphatidylcholine (PC) (36:5; 38:6; 40:6; o38:6; p40:6), PE (p36:5; p38:6), lysophosphatidylcholine (LPC) (22:6), hydroxyproline, and CMPF.

### 3.3. Associations between Dietary Consumption and Plasma Metabolites

We examined the associations of meat and seafood consumption with 42 differential metabolites in the study population (*n* = 270) by conducting Pearson correlation analysis with adjustments for age, gender, BMI, fruits, whole grains, and soy products. Significance was defined as the Bonferroni-corrected level of *p* < 6.0 × 10^−4^ (2 food groups × 42 differential metabolites, at the 0.05 level). According to the analysis, a diet high in meat was associated with higher levels of AA- and DHA-content glycerophospholipids; a diet high in seafood was associated with higher concentrations of EPA, DHA, and their-content glycerophospholipids, as well as d-glucose and three amino acids (hydroxyproline, lysine, and valine); whereas increasing the consumption of meat and seafood was associated with lower non-AA/EPA/DHA-content glycerophospholipids (Table 3). Furthermore, we evaluated the associations between consumption of the 12 typical food groups in the Singapore Chinese diet (including red meat, poultry, fish, shellfish, vegetables, fruits, whole grains, refined grains, soy products, dairy, eggs, nuts, and seeds) and the concentrations of 42 differential metabolites in the study population (*n* = 270) with adjustments for age, gender, and BMI for post hoc analysis. We identified 32 correlations between food groups and differential metabolites that were significant at the Bonferroni-corrected level of *p* < 1.0 × 10^−4^ (*p* = 0.05/(12 food groups × 42 differential metabolites)) (Table 3). These correlations represented six food groups including red meat, poultry, fish, shellfish, soy products, and dairy, but most of the findings were for fish and soy products. The strongest correlations (those with *p *< 1.0 × 10^−10^) were found between fish consumption and DHA (*r* = 0.428), DHA-content glycerophospholipids (PC (38:6; 40:6; p40:6), PE (p38:6)) (*r* > 0.400), and CMPF (*r* = 0.411). In addition to fish, we found that DHA-content glycerophospholipids were also correlated with poultry (PC (o38:6; 38:6)) and soy products (PC (38:6; 40:6; p40:6; o38:6), LPE (18:2), PE (p38:6)).

### 3.4. Associations of Plasma Metabolites with Clinical Characteristics

Table 4 shows the correlations between plasma metabolites and clinical characteristics (including fasting glucose, fasting insulin, creatinine, total cholesterol, triglyceride (TG), high density lipoprotein (HDL), low density lipoprotein (LDL), and C-reactive protein). As shown, there were 89 significant correlations at the Bonferroni-corrected level of *p* < 1.5 × 10^−4^ (*p* = 0.05/(8 clinical characteristics × 42 differential metabolites)). Notably, fasting glucose was positively correlated with plasma d-glucose, hydroxyproline, valine, CMPF, EPA, and DHA, with *r* ranging from 0.235 to 0.638. Total cholesterol, TG, HDL, and LDL were mainly correlated with AA, DHA, and their -content glycerophospholipids. Additional, LDL was positively correlated with lysine and valine, whereas HDL was negatively correlated with them. There was no significant correlation observed between C-reactive protein and plasma metabolites.

## 4. Discussion

In the context of the increasing consumption of meat and seafood and the increasing prevalence of diet-related chronic diseases in Asian populations, we investigated habitual meat or seafood consumption in relation to plasma metabolic profiles in a Singapore Chinese population. The participants were divided into four dietary groups according to the amounts of meat and seafood consumed. We identified 42 metabolites that significantly differed between the four dietary groups, including 31 glycerophospholipids, four fatty acids, six amino acids, and one carbohydrate. High meat or seafood consumers had higher concentrations of AA/EPA/DHA-content glycerophospholipids, fatty acids (AA, EPA, DHA, and CMPF), essential amino acids (lysine, threonine, tryptophan, and valine), and d-glucose as compared with low consumers of these animal products. In contrast, high meat and seafood consumers had lower concentrations of non-AA/EPA/DHA-content glycerophospholipids and glycine. Our analysis of the associations between specific foods and plasma metabolites, confirmed the previously reported associations between fish consumption and EPA, DHA, and CMPF [40]. In addition, we observed novel associations between poultry consumption and two DHA-content glycerophospholipids (PC (38:6; o38:6)), and between shellfish consumption and an AA-content glycerophospholipid (PE (p36:4)), which may reflect the co-consumption of meat and seafood in our population. With regard to clinical parameters, several plasma metabolites (hydroxyproline, valine, EPA, DHA, and CMPF) were associated with fasting glucose concentrations.

Omega-6 (*n*-6) and omega-3 (*n*-3) polyunsaturated fatty acids (PUFAs) regulate a wide range of functions in the body, including blood pressure, blood clotting, and the development and functioning of the brain and nervous systems [41]. Moreover, they play important roles in the regulation of inflammation as precursors of inflammatory mediators, termed “eicosanoids” [42]. In this study, we found that the total AA (*n*-6), EPA (*n*-3) and DHA (*n*-3) were higher in high meat or seafood consumers than LMLS consumers. Of these, EPA and DHA were significantly associated with dietary exposure to fish. Our findings are consistent with biosynthesis pathways and dietary sources of these two PUFAs. The body can convert alpha-linolenic acid to EPA and DHA [43], and the body can also obtain them directly from dietary sources. Analyses of food composition have shown that marine fish are the main source of EPA and DHA [44], which supports our findings. In addition to PUFAs, an endogenous metabolite of furan fatty acids, CMPF, was found to be higher in high meat or seafood consumers than LMLS consumers, and its level was directly associated with dietary exposure to fish. This is consistent with previous studies that show the consumption of fish increases circulating CMPF levels [45].

Along with the accumulation of AA, EPA, and DHA, higher levels of their-content glycerophospholipids were also found in high meat or seafood consumers than LMLS consumers. PC and PE species account for greater than 50% of the total phospholipids in eukaryotic membranes [46]. Besides their roles as structural components of membranes, they have several other functions. PC is an important source for the formation of second messengers and lipid mediators, and PE plays essential roles in autophagy, cell division, and protein folding, and represents a precursor for the synthesis of several protein modifications [47]. Both PC and PE are provided by the diet (mainly from animal products) and by de novo biosynthesis [20]. The de novo biosynthesis of PC and PE in eukaryotic cells proceeds mainly via the CDP-choline and CDP-ethanolamine branches of the Kennedy pathway [46]. Of the two branches of the Kennedy pathway, one requires choline (mainly found in animal products), and the other one needs ethanolamine (mainly from dietary sources and a small amount from sphingolipid catabolism). In this study, we observed strong associations between AA/EPA/DHA-content PC and PE species with dietary exposure to poultry, fish, shellfish, and soy products, which are consistent with the synthesis pathways and dietary sources of these metabolites.

Besides various lipid species, a series of amino acids such as hydroxyproline, lysine, threonine, tryptophan, and valine were found to be significantly higher in high meat or seafood consumers than LMLS consumers. Lysine, threonine, tryptophan, and valine are essential amino acids, and their levels in plasma were seriously affected by food intake. Good sources of these amino acids are high-protein foods such as animal products [48,49]. Hydroxyproline is a non-proteinogenic amino acid, which is produced by the hydroxylation of proline. Although hydroxyproline has been traditionally considered to have little nutritional significance, it is now recognized as a substrate for the synthesis of glycine, pyruvate, and glucose. Proline and hydroxyproline are most abundant in salmon proteins [50]. These studies are in line with our findings that hydroxyproline was strongly associated with dietary exposure to fish.

Additionally, we found that plasma d-glucose levels in high meat or seafood consumers were higher than that in LMLS consumers. Previous studies suggest that red meat intake is positively associated with glucose metabolism [6,51], whereas the association between fish intake and glucose levels is controversial [52,53]. However, in the present study we noticed that d-glucose showed stronger association with high fish intake than high red meat consumption. The findings were consistent with the clinical characteristics of participants that high seafood consumers (i.e., HMHS and LMHS) had higher fasting blood glucose concentrations than HMLS and LMLS consumers (Table 1). Furthermore, it was found that these plasma metabolites (such as EPA, DHA, CMPF, d-glucose, and hydroxyproline) that were associated with fish consumption were also associated with fasting blood glucose concentrations (Table 3 and Table 4). These findings indicated potential putative effects of high fish consumption in glucose metabolism. Further research is required to verify this observation.

This study is among the first to investigate the plasma metabolic profiles in a Chinese population with different patterns of meat and seafood consumption, using both untargeted and targeted metabolomics strategies. Here, some limitations of our study still need to be acknowledged. First, although there was no significant difference in total energy intake among the four dietary groups, we cannot fully exclude confounders by other dietary factors in analyzing differential metabolites and associations, as there were also some differences in the consumption of fruit, whole grains, and soy products amongst participants. Second, within the untargeted analysis, glycerophospholipids were not confirmed by using commercial standards, yet they were defined by their unique mass fragmental patterns and previous reports [54,55]. Third, our study had a few missing data points on clinical characteristics (fasting glucose, LDL, and C-reactive protein) of participants as shown in Table 1. Lastly, our findings were observed in a single cohort and should be validated in an independent population of ethnic Chinese.

## 5. Conclusions

In summary, we identified 42 plasma metabolites that differed according to meat and seafood consumption in an ethnic Chinese population. High meat or seafood consumers had higher levels of AA, EPA, DHA, and their-content glycerophospholipids, as well as amino acids, CMPF, and d-glucose. These findings demonstrate a significant influence of dietary patterns on plasma metabolic profiles. Furthermore, we identified significant correlations between several plasma metabolites and fasting glucose concentrations. Further research may elucidate whether these compounds or related biological pathways contribute to the relationship between the consumption of animal products and the risk of type 2 diabetes.

## Figures and Tables

**Table 1:** Demographic and clinical characteristics of 270 participants by diet group: high meat and high seafood (HMHS), high meat and low seafood (HMLS), low meat and high seafood (LMHS), and low meat and low seafood (LMLS) consumers.

Characteristics ^a^	HMHS Consumers (*n* = 60)	HMLS Consumers (*n* = 64)	LMHS Consumers (*n* = 60)	LMLS Consumers (*n* = 86)	*p* ^b^
Age (year)	50.18 ± 12.43 ^c^	50.19 ± 13.37	53.03 ± 11.64	49.28 ± 11.29	0.310
Sex *n* (%)					0.735
Male	33 (55.0%)	29 (45.3%)	31 (51.7%)	42 (48.8%)	
Female	27 (45.0%)	35 (54.7%)	29 (48.3%)	44 (51.2%)	
Alcohol consumption status *n* (%)					0.051
Abstainer	40 (66.7%)	47 (73.4%)	30 (50.0%)	53 (61.6%)	
Consumer	20 (33.3%)	17 (26.6%)	30 (50.0%)	33 (38.4%)	
BMI (kg/m^2^)	23.41 ± 3.88	23.17 ± 3.62	23.83 ± 4.15	22.57 ± 3.82	0.255
Fasting glucose (mmol/L)	5.14 ± 1.32 *	5.07 ± 1.48	5.27 ± 1.36 *	4.71 ± 0.58	0.036
Fasting insulin (mU/L)	7.55 ± 4.10	7.51 ± 6.15	8.11 ± 7.16	7.65 ± 13.64	0.982
Creatinine (µmol/L)	78.62 ± 19.64	75.36 ±15.21	79.67 ± 20.21	79.30 ± 16.82	0.503
Total cholesterol (mmol/L)	5.21 ± 0.98	5.23 ± 1.07	5.14 ± 0.94	5.02 ± 0.88	0.541
TG (mmol/L)	1.38 ± 0.76	1.19 ± 0.61	1.20 ± 0.65	1.39 ± 1.32	0.401
HDL (mmol/L)	1.33 ± 0.32	1.45 ± 0.35	1.40 ± 0.34	1.44 ± 0.38	0.211
LDL (mmol/L)	3.25 ± 0.86	3.24 ± 0.92	3.20 ± 0.79	2.97 ± 0.67	0.107
C-reactive protein (mg/L)	1.79 ± 2.62	3.84 ± 10.69	1.33 ± 1.34	1.98 ± 4.18	0.092

TG: triglyceride; HDL: high density lipoprotein; LDL: low density lipoprotein. ^a ^Information was missing for some participants: fasting glucose, *n* = 6; LDL-cholesterol, *n* = 3; C-reactive protein, *n* = 7; ^b^ One-way ANOVA for continuous variables, and by chi-square test for categorical variables; ^c ^Mean ± SD (all such values). * *p* < 0.05 compared to the LMLS group, Student’s *t*-test.

**Table 2:** Forty-two metabolites for which the plasma concentration differed significantly among high meat and high seafood (HMHS, *n* = 60), high meat and low seafood (HMLS, *n* = 64), low meat and high seafood (LMHS, *n* = 60), and low meat and low seafood (LMLS, *n* = 86) consumers.

Metabolite	Candidate	Fold Differences			Area under the Curve (AUC) (95% CI)		
		HMHS/LMLS	HMLS/LMLS	LMHS/LMLS	HMHS vs. LMLS	HMLS vs. LMLS	LMHS vs. LMLS
**Untargeted analysis**							
*AA-content glycerophospholipids*							
PC (36:4)	PC (20:4/16:0)	1.08 *	1.10 **	1.07	0.60 (0.51, 0.69)	0.62 (0.53, 0.71)	0.57 (0.47, 0.66)
PC (38:4)	PC (18:0/20:4)	1.11 *	1.14 **	1.07	0.61 (0.51, 0.70)	0.62 (0.53, 0.71)	0.56 (0.46, 0.65)
PC (p36:4)	PC (20:4/P-16:0)	1.15 **	1.18 **	1.08	0.66 (0.57, 0.75)	0.69 (0.60, 0.77)	0.58 (0.49, 0.67)
PC (o36:4)	PC (O-16:0/20:4)	1.14 **	1.15 **	1.10 *	0.64 (0.55, 0.73)	0.65 (0.57, 0.74)	0.60 (0.51, 0.70)
PC (o38:5)	PC (O-18:1/20:4)	1.08 *	1.11 **	1.04	0.59 (0.50, 0.68)	0.63 (0.54, 0.72)	0.55 (0.46, 0.65)
PE (p36:4)	PE (P-16:0/20:4)	1.31 **	1.26 **	1.17 *	0.72 (0.64, 0.81)	0.69 (0.61, 0.78)	0.64 (0.55, 0.73)
PE (p38:4)	PE (P-18:0/20:4)	1.29 **	1.29 **	1.21 *	0.69 (0.60, 0.78)	0.69 (0.61, 0.78)	0.65 (0.57, 0.74)
PE (p38:5)	PE (P-18:1/20:4)	1.25 **	1.24 **	1.17 *	0.68 (0.59, 0.77)	0.67 (0.59, 0.76)	0.64 (0.55, 0.73)
*EPA-content glycerophospholipids*							
PC (36:5)	PC (16:0/20:5)	1.64 **	1.31 *	1.83 **	0.74 (0.66, 0.82)	0.61 (0.53, 0.70)	0.78 (0.71, 0.86)
PE (p36:5)	PE (P-16:0/20:5)	1.92 **	1.52 **	2.16 **	0.76 (0.68, 0.84)	0.66 (0.57, 0.74)	0.77 (0.70, 0.85)
*DHA-content glycerophospholipids*							
LPC (22:6)		1.45 **	1.29 **	1.42 **	0.69 (0.60, 0.77)	0.65 (0.56, 0.74)	0.71 (0.63, 0.80)
PC (38:6)	PC (22:6/16:0)	1.36 **	1.26 **	1.35 **	0.77 (0.69, 0.84)	0.70 (0.62, 0.78)	0.75 (0.68, 0.83)
PC (40:6)	PC (22:6/18:0)	1.41 **	1.27 **	1.38 **	0.76 (0.68, 0.84)	0.67 (0.59, 0.76)	0.73 (0.65, 0.81)
PC (40:7)	PC (18:1/22:6)	1.16 *	1.18 **	1.23 **	0.61 (0.52, 0.70)	0.63 (0.55, 0.72)	0.65 (0.56, 0.74)
PC (o38:6)	PC (O-16:0/22:6)	1.32 **	1.28 **	1.36 **	0.74 (0.65, 0.82)	0.73 (0.65, 0.81)	0.73 (0.65, 0.81)
PC (p40:6)	PC (P-18:0/22:6)	1.36 **	1.30 **	1.42 **	0.73 (0.65, 0.81)	0.70 (0.62, 0.78)	0.74 (0.66, 0.82)
LPE (22:6)		1.27 **	1.24 **	1.26 **	0.70 (0.62, 0.79)	0.68 (0.59, 0.76)	0.70 (0.62, 0.79)
PE (38:6)	PE (22:6/16:0)	1.40 **	1.31 **	1.38 **	0.69 (0.60, 0.77)	0.68 (0.60, 0.77)	0.72 (0.63, 0.80)
PE (40:6)	PE (22:6/18:0)	1.53 **	1.34 **	1.42 **	0.73 (0.65, 0.81)	0.69 (0.60, 0.77)	0.71 (0.63, 0.79)
PE (40:9)	PE (22:6/18:3)	1.40 **	1.28 **	1.35 **	0.68 (0.59, 0.76)	0.66 (0.57, 0.74)	0.68 (0.59, 0.76)
PE (p38:6)	PE (P-16:0/22:6)	1.44 **	1.30 **	1.41 **	0.78 (0.70, 0.85)	0.70 (0.62, 0.79)	0.75 (0.67, 0.83)
*Non-AA/EPA/DHA-content Glycerophospholipids*							
PC (34:2)	PC (18:2/16:0)	0.94*	0.92**	0.96	0.62 (0.53, 0.71)	0.63 (0.54, 0.72)	0.57 (0.47, 0.66)
PC (36:2)	PC (18:1/18:1)	0.81 **	0.87	0.82 **	0.64 (0.54, 0.73)	0.59 (0.49, 0.68)	0.62 (0.52, 0.71)
PC (36:4)	PC (18:2/18:2)	0.90 **	0.96	0.91 **	0.66 (0.57, 0.75)	0.58 (0.49, 0.67)	0.62 (0.53, 0.72)
PC (o34:1)	PC (O-16:0/18:1)	0.93 **	0.93 **	0.95 *	0.64 (0.55, 0.73)	0.64 (0.55, 0.73)	0.59 (0.50, 0.69)
LPE (18:1)		0.82 *	0.82 *	0.87	0.63 (0.53, 0.72)	0.61 (0.52, 0.70)	0.58 (0.48, 0.67)
LPE (18:2)		0.70 **	0.71 **	0.75 **	0.67 (0.58, 0.76)	0.66 (0.58, 0.75)	0.63 (0.54, 0.73)
PE (36:3)	PE (18:2/18:1)	0.56 **	0.65 **	0.65 **	0.66 (0.57, 0.75)	0.63 (0.54, 0.72)	0.61 (0.52, 0.70)
PI (34:2)	PI (18:2/16:0)	0.78 **	0.81 **	0.87 *	0.69 (0.60, 0.78)	0.65 (0.56, 0.74)	0.59 (0.50, 0.69)
PI (36:2)	PI (18:2/18:0)	0.80 *	0.84 *	0.91	0.62 (0.53, 0.71)	0.59 (0.50, 0.68)	0.51 (0.41, 0.60)
PI (38:3)	PI (20:3/18:0)	0.72 **	0.81 *	0.78 **	0.66 (0.57, 0.75)	0.58 (0.49, 0.68)	0.60 (0.51, 0.70)
*Other*							
d-Glucose		1.09 **	1.04	1.10 **	0.66 (0.56, 0.75)	0.58 (0.49, 0.68)	0.70 (0.61, 0.79)
CMPF		2.10 **	1.32	2.11 **	0.77 (0.69, 0.84)	0.64 (0.55, 0.72)	0.77 (0.70, 0.85)
**Targeted analysis**							
*Amino acids*							
Glycine		0.89 *	0.98	0.98	0.64 (0.55, 0.73)	0.51 (0.42, 0.61)	0.52 (0.42, 0.61)
Hydroxyproline		1.43 **	1.19	1.46 **	0.71 (0.63, 0.80)	0.64 (0.55, 0.73)	0.73 (0.65, 0.81)
Lysine		1.16 **	1.10**	1.15 **	0.64 (0.54, 0.73)	0.62 (0.53, 0.71)	0.65 (0.57, 0.74)
Threonine		1.08 *	0.97	1.01	0.63 (0.53, 0.72)	0.52 (0.43, 0.61)	0.51 (0.42, 0.61)
Tryptophan		1.07 *	0.97	1.04	0.63 (0.54, 0.72)	0.52 (0.43, 0.61)	0.58 (0.48, 0.67)
Valine		1.07 *	1.05	1.10 **	0.60 (0.51, 0.69)	0.57 (0.48, 0.66)	0.66 (0.57, 0.75)
*Total fatty acids*							
AA		1.26 **	1.22 **	1.10	0.67 (0.58, 0.76)	0.64 (0.55, 0.73)	0.59 (0.5, 0.68)
EPA		1.50 **	1.24	1.60 **	0.74 (0.66, 0.82)	0.61 (0.52, 0.70)	0.78 (0.7, 0.85)
DHA		1.78 **	1.40 **	1.63 **	0.79 (0.72, 0.86)	0.71 (0.63, 0.79)	0.77 (0.7, 0.85)

AA, arachidonic acid; PC, phosphatidylcholine; PE, phosphatidylethanolamine; EPA, eicosapentaenoic acid, DHA, docosahexaenoic acid; LPC, lysophosphatidylcholine; LPE, lysophosphatidylethanolamine; PI, phosphatidylinositol; CMPF, 3-carboxy-4-methyl-5-propyl-2-furanpropanoic acid. (o), ether-linked phospholipids; (p) plasmalogen-based phospholipids. * *p* < 0.05; ** *p* < 0.01, Student’s *t*-test.

**Table 3:** Top metabolites associated with food groups in the study population (*n* = 270).

Food Groups	Metabolites	Class	Correlation (*r*)	*p*
Meat ^a^	PC (p36:4)	AA-content GP	0.254	2.43 × 10^−05^
	PE (p36:4)	AA-content GP	0.235	9.42 × 10^−05^
	PC (o36:4)	AA-content GP	0.215	3.73 × 10^−04^
	PC (38:6)	DHA-content GP	0.263	1.22 × 10^−05^
	PC (40:6)	DHA-content GP	0.223	2.24 × 10^−04^
	PC (o38:6)	DHA-content GP	0.216	3.49 × 10^−04^
	LPE (18:2)	Non-AA/EPA/DHA-content GP	−0.280	3.01 × 10^−06^
	PI (34:2)	Non-AA/EPA/DHA-content GP	−0.246	4.44 × 10^−05^
	PE (36:3)	Non-AA/EPA/DHA-content GP	−0.241	6.13 × 10^−05^
Seafood ^a^	Hydroxyproline	Amino acids	0.321	6.91 × 10^−08^
	Valine	Amino acids	0.247	3.97 × 10^−05^
	Lysine	Amino acids	0.240	6.92 × 10^−05^
	d-Glucose	Carbohydrates	0.252	2.70 × 10^−05^
	CMPF	Fatty acids	0.399	9.26 × 10^−12^
	DHA	Fatty acids	0.421	5.13 × 10^−13^
	EPA	Fatty acids	0.285	1.91 × 10^−06^
	PC (38:6)	DHA-content GP	0.442	2.62 × 10^−14^
	PE (p38:6)	DHA-content GP	0.421	5.37 × 10^−13^
	PC (40:6)	DHA-content GP	0.415	1.14 × 10^−12^
	PC (p40:6)	DHA-content GP	0.405	4.68 × 10^−12^
	PC (o38:6)	DHA-content GP	0.377	1.46 × 10^−10^
	LPC (22:6)	DHA-content GP	0.343	7.35 × 10^−09^
	PE (40:6)	DHA-content GP	0.304	3.64 × 10^−07^
	LPE (22:6)	DHA-content GP	0.293	9.40 × 10^−07^
	PE (38:6)	DHA-content GP	0.258	1.83 × 10^−05^
	PE (40:9)	DHA-content GP	0.255	2.27 × 10^−05^
	PE (p36:5)	EPA-content GP	0.387	4.65 × 10^−11^
	PC (36:5)	EPA-content GP	0.341	9.22 × 10^−09^
	PI (38:3)	Non-AA/EPA/DHA-content GP	−0.238	7.84 × 10^−05^
	LPE (18:2)	Non-AA/EPA/DHA-content GP	−0.224	2.05 × 10^−04^
Red meat ^b^	LPE (18:2)	Non-AA/EPA/DHA-content GP	−0.235	9.84 × 10^−05^
Poultry ^b^	PC (p36:4)	AA-content GP	0.262	1.30 × 10^−05^
	PC (o38:6)	DHA-content GP	0.250	3.19 × 10^−05^
	PC (38:6)	DHA-content GP	0.235	9.36 × 10^−05^
Fish ^b^	Hydroxyproline	Amino acids	0.310	2.03 × 10^−07^
	d-Glucose	Carbohydrates	0.252	2.83 × 10^−05^
	CMPF	Fatty acids	0.411	1.94 × 10^−12^
	DHA	Fatty acids	0.428	1.93 × 10^−13^
	EPA	Fatty acids	0.299	5.77 × 10^−07^
	PC (38:6)	DHA-content GP	0.448	9.82 × 10^−15^
	PC (40:6)	DHA-content GP	0.426	2.39 × 10^−13^
	PC (p40:6)	DHA-content GP	0.414	1.31 × 10^−12^
	PE (p38:6)	DHA-content GP	0.405	4.19 × 10^−12^
	LPC (22:6)	DHA-content GP	0.371	2.94 × 10^−10^
	PC (o38:6)	DHA-content GP	0.369	3.98 × 10^−10^
	LPE (22:6)	DHA-content GP	0.301	4.56 × 10^−07^
	PE (40:6)	DHA-content GP	0.297	6.67 × 10^−07^
	PE (40:9)	DHA-content GP	0.251	2.94 × 10^−05^
	PE (38:6)	DHA-content GP	0.251	3.10 × 10^−05^
	PE (p36:5)	EPA-content GP	0.382	8.75 × 10^−11^
	PC (36:5)	EPA-content GP	0.356	1.72 × 10^−09^
Shellfish ^b^	PE (p36:4)	AA-content GP	0.243	5.58 × 10^−05^
Vegetables ^b^	-			
Fruits ^b^	-			
Whole grains ^b^	-			
Refined grains ^b^	-			
Soy products ^b^	PC (o38:5)	AA-content GP	−0.247	4.06 × 10^−05^
	PC (38:6)	DHA-content GP	−0.308	2.51 × 10^−07^
	PC (40:6)	DHA-content GP	−0.304	3.37 × 10^−07^
	PC (p40:6)	DHA-content GP	−0.289	1.35 × 10^−06^
	PC (o38:6)	DHA-content GP	−0.277	3.94 × 10^−06^
	PE (p38:6)	DHA-content GP	−0.252	2.85 × 10^−05^
	LPE (18:2)	DHA-content GP	0.236	9.15 × 10^−05^
	PE (p36:5)	EPA-content GP	−0.236	9.17 × 10^−05^
	PI (38:3)	Non-AA/EPA/DHA-content GP	0.279	3.11 × 10^−06^
Dairy ^b^	Valine	Amino acids	−0.242	5.67 × 10^−05^
Eggs ^b^	-			
Nuts and seeds ^b^	-			

LPC, lysophosphatidylcholine; PC, phosphatidylcholine; LPE, lysophosphatidylethanolamine; PE, phosphatidylethanolamine; EPA, eicosapentaenoic acid, DHA, docosahexaenoic acid; PI, phosphatidylinositol; CMPF, 3-carboxy-4-methyl-5-propyl-2-furanpropanoic acid. (o), ether-linked phospholipids; (p) plasmalogen-based phospholipids. AA, arachidonic acid; GP, glycerophospholipids. ^a^ Pearson correlation analysis with adjustments for age, gender, BMI, fruits, whole grains, and soy products. Significance was defined as the Bonferroni-corrected level of *p* < 6.0 × 10^−4^ (2 food groups × 42 differential metabolites, at the 0.05 level). ^b^ Pearson correlation analysis with adjustments for age, gender, and BMI. Significance was defined as the Bonferroni-corrected level of *p* < 1.0 × 10^−4^ (12 food groups × 42 differential metabolites, at the 0.05 level).

**Table 4:** Top metabolites associated with clinical characteristics in the study population (*n* = 270) ^a^.

Clinical Characteristics	Metabolites	Class	Correlation (*r*)	*p*
Fasting glucose	d-Glucose	Carbohydrates	0.638	1.61 × 10^−31^
	Hydroxyproline	Amino acids	0.248	4.60 × 10^−05^
	Valine	Amino acids	0.235	1.15 × 10^−04^
	CMPF	Fatty acids	0.401	1.28 × 10^−11^
	DHA	Fatty acids	0.284	2.72 × 10^−06^
	EPA	Fatty acids	0.236	1.08 × 10^−04^
Fasting insulin	d-Glucose	Carbohydrates	0.301	4.79 × 10^−07^
	Valine	Amino acids	0.267	8.99 × 10^−06^
Creatinine	Valine	Amino acids	0.335	1.67 × 10^−08^
	Hydroxyproline	Amino acids	0.332	2.18 × 10^−08^
	Tryptophan	Amino acids	0.247	4.00 × 10^−05^
Total cholesterol	AA	Fatty acids	0.520	3.99 × 10^−20^
	DHA	Fatty acids	0.431	1.22 × 10^−13^
	PC (38:4)	AA-content GP	0.416	9.77 × 10^−13^
	PC (o36:4)	AA-content GP	0.332	2.26 × 10^−08^
	PC (o38:5)	AA-content GP	0.529	7.50 × 10^−21^
	PC (p36:4)	AA-content GP	0.385	6.01 × 10^−11^
	PE (p36:4)	AA-content GP	0.298	6.32 × 10^−07^
	PE (p38:4)	AA-content GP	0.434	8.26 × 10^−14^
	PE (p38:5)	AA-content GP	0.476	1.08 × 10^−16^
	LPE (22:6)	DHA-content GP	0.250	3.34 × 10^−05^
	PC (38:6)	DHA-content GP	0.409	2.57 × 10^−12^
	PC (40:6)	DHA-content GP	0.436	5.88 × 10^−14^
	PC (40:7)	DHA-content GP	0.350	3.28 × 10^−09^
	PC (o38:6)	DHA-content GP	0.266	9.51 × 10^−06^
	PC (p40:6)	DHA-content GP	0.310	2.00 × 10^−07^
	PE (38:6)	DHA-content GP	0.360	1.09 × 10^−09^
	PE (40:6)	DHA-content GP	0.386	5.13 × 10^−11^
	PE (40:9)	DHA-content GP	0.294	9.11 × 10^−07^
	PE (p38:6)	DHA-content GP	0.561	7.99 × 10^−24^
	PC (34:2)	Non-AA/EPA/DHA-content GP	0.603	4.17 × 10^−28^
	PC (36:2)	Non-AA/EPA/DHA-content GP	0.376	1.62 × 10^−10^
	PC (36:4)	Non-AA/EPA/DHA-content GP	0.436	6.29 × 10^−14^
	PC (o34:1)	Non-AA/EPA/DHA-content GP	0.524	1.92 × 10^−20^
	PE (36:3)	Non-AA/EPA/DHA-content GP	0.274	4.97 × 10^−06^
	PI (34:2)	Non-AA/EPA/DHA-content GP	0.336	1.56 × 10^−08^
	PI (36:2)	Non-AA/EPA/DHA-content GP	0.572	7.78 × 10^−25^
	PI (38:3)	Non-AA/EPA/DHA-content GP	0.299	5.57 × 10^−07^
TG	Lysine	Amino acids	0.256	2.06 × 10^−05^
	Valine	Amino acids	0.323	5.81 × 10^−08^
	AA	Fatty acids	0.406	4.00 × 10^−12^
	DHA	Fatty acids	0.304	3.37 × 10^−07^
	PC (38:4)	AA-content GP	0.239	7.34 × 10^−05^
	PE (38:6)	DHA-content GP	0.338	1.28 × 10^−08^
	PE (40:6)	DHA-content GP	0.490	1.13 × 10^−17^
	PE (40:9)	DHA-content GP	0.309	2.17 × 10^−07^
	LPE (18:1)	Non-AA/EPA/DHA-content GP	0.247	4.15 × 10^−05^
	PC (34:2)	Non-AA/EPA/DHA-content GP	0.367	4.81 × 10^−10^
	PC (36:2)	Non-AA/EPA/DHA-content GP	0.585	3.64 × 10^−26^
	PC (36:4)	Non-AA/EPA/DHA-content GP	0.302	4.19 × 10^−07^
	PC (o34:1)	Non-AA/EPA/DHA-content GP	0.318	9.70 × 10^−08^
	PE (36:3)	Non-AA/EPA/DHA-content GP	0.566	2.79 × 10^−24^
	PI (34:2)	Non-AA/EPA/DHA-content GP	0.511	2.11 × 10^−19^
	PI (36:2)	Non-AA/EPA/DHA-content GP	0.642	1.04 × 10^−32^
	PI (38:3)	Non-AA/EPA/DHA-content GP	0.506	5.63 × 10^−19^
HDL	Lysine	Amino acids	−0.315	1.22 × 10^−07^
	Valine	Amino acids	−0.482	3.99 × 10^−17^
	PC (o36:4)	AA-content GP	0.233	1.09 × 10^−04^
	PC (o38:5)	AA-content GP	0.410	2.34 × 10^−12^
	PC (p36:4)	AA-content GP	0.298	6.01 × 10^−07^
	PE (p38:4)	AA-content GP	0.235	9.58 × 10^−05^
	PE (p38:5)	AA-content GP	0.261	1.35 × 10^−05^
	PC (40:7)	DHA-content GP	0.446	1.37 × 10^−14^
	PC (o38:6)	DHA-content GP	0.368	4.33 × 10^−10^
	PC (p40:6)	DHA-content GP	0.390	2.88 × 10^−11^
	PE (p38:6)	DHA-content GP	0.235	9.95 × 10^−05^
	PC (34:2)	Non-AA/EPA/DHA-content GP	0.378	1.31 × 10^−10^
	PC (o34:1)	Non-AA/EPA/DHA-content GP	0.343	7.09 × 10^−09^
	PC (36:4)	Non-AA/EPA/DHA-content GP	0.549	1.19 × 10^−22^
LDL	AA	Fatty acids	0.423	5.06 × 10^−13^
	DHA	Fatty acids	0.361	1.28 × 10^−09^
	PC (38:4)	AA-content GP	0.329	3.75 × 10^−08^
	PC (o36:4)	AA-content GP	0.315	1.48 × 10^−07^
	PC (o38:5)	AA-content GP	0.438	6.40 × 10^−14^
	PC (p36:4)	AA-content GP	0.355	2.28 × 10^−09^
	PE (p36:4)	AA-content GP	0.282	2.86 × 10^−06^
	PE (p38:4)	AA-content GP	0.344	7.91 × 10^−09^
	PE (p38:5)	AA-content GP	0.379	1.57 × 10^−10^
	PC (38:6)	DHA-content GP	0.350	4.32 × 10^−09^
	PC (40:6)	DHA-content GP	0.380	1.40 × 10^−10^
	PC (o38:6)	DHA-content GP	0.256	2.23 × 10^−05^
	PC (p40:6)	DHA-content GP	0.289	1.60 × 10^−06^
	PE (40:6)	DHA-content GP	0.269	8.35 × 10^−06^
	PE (p38:6)	DHA-content GP	0.485	3.52 × 10^−17^
	PC (34:2)	Non-AA/EPA/DHA-content GP	0.428	2.71 × 10^−13^
	PC (36:2)	Non-AA/EPA/DHA-content GP	0.252	3.08 × 10^−05^
	PC (36:4)	Non-AA/EPA/DHA-content GP	0.338	1.55 × 10^−08^
	PC (o34:1)	Non-AA/EPA/DHA-content GP	0.361	1.28 × 10^−09^
	PI (36:2)	Non-AA/EPA/DHA-content GP	0.520	6.75 × 10^−20^
C-reactive protein	-			

PC, phosphatidylcholine; LPE, lysophosphatidylethanolamine; PE, phosphatidylethanolamine; PI, phosphatidylinositol; AA, arachidonic acid; EPA, eicosapentaenoic acid, DHA, docosahexaenoic acid; CMPF, 3-carboxy-4-methyl-5-propyl-2-furanpropanoic acid. (o), ether-linked phospholipids; (p) plasmalogen-based phospholipids. GP, glycerophospholipids. ^a^ Pearson correlation analysis with adjustments for age, gender, and BMI. Significance was defined as the Bonferroni-corrected level of *p* < 1.5 × 10^−4^ (8 clinical characteristics × 42 differential metabolites, at the 0.05 level).

## Supplementary Materials

The following are available online at www.mdpi.com/2072-6643/9/7/683/s1, Table S1: Reproducibility evaluation of LC-MS and GC-MS based on selected peaks in quality control samples, Table S2: Dietary intake (g/2000 kcal, Mean ± SD) of 270 participants by diet groups, Table S3: Optimized MRM conditions for determination of 16 amino acids, Table S4: Optimized MRM conditions for determination of 19 fatty acid methyl esters, Table S5: AUC values of 42 metabolites by comparing the top and the bottom quintiles of meat and seafood consumption, Figure S1: The association between food consumption and plasma metabolic profile, Figure S2: Principal component analysis (PCA) score scatter plots of all plasma (grey dots, *n* = 270) and QC (green dots, *n* = 27) samples, Figure S3: Fold differences of plasma metabolites in HMHS, HMLS and LMHS consumers by comparison with LMLS consumers.
