# Supplementary material for: Meat and Seafood Consumption in Relation to Plasma Metabolic Profiles in a Chinese Population: A Combined Untargeted and Targeted Metabolomics Study
Source: Nutrients. 2017 Jun 30;9(7):683. doi: 10.3390/nu9070683 (PMC5537798; doi:10.3390/nu9070683)
Supplement: Supplementary file 1 [file nutrients-09-00683-s001.pdf]

Table S1. Reproducibility evaluation of LC-MS and GC-MS based on selected peaks in quality control samples.

Peak	LC-MS, positive ion mode			Peak	LC-MS, negative ion mode			Peak	GC-MS		
No.	m/z	R.T.(min)	RSD [a] (100%)	No.	m/z	R.T.(min)	RSD [a] (100%)	No.	m/z	R.T.(min)	RSD [a] (100%)
1	231.06	0.8	5.8	1	130.09	0.6	7.5	1	116.09	4.8	8.6
2	144.08	1.5	9.2	2	203.08	1.4	10.3	2	151.07	6.0	10.2
3	388.25	2.9	12.1	3	187.00	2.5	6.9	3	142.11	7.8	6.9
4	585.27	4.0	10.9	4	475.19	4.6	7.9	4	204.13	8.8	9.2
5	465.03	5.8	8.6	5	381.07	5.1	12.8	5	258.10	11.2	7.7
6	620.44	6.8	7.7	6	448.31	6.1	16.4	6	295.08	15.0	9.6
7	546.35	8.0	13.5	7	568.36	7.8	15.7	7	84.09	17.3	10.2
8	640.54	10.0	9.2	8	595.49	9.9	13.4	8	339.27	20.1	6.1
9	748.53	11.9	14.8	9	788.58	12.7	9.8	9	285.19	23.0	10.4
10	810.60	13.4	15.9	10	656.57	14.0	14.5	10	316.20	26.3	11.6

[a] RSD: relative standard deviation of peak areas.

Table S2. Dietary intake (g/2000 kcal, Mean ± SD) of 270 participants by diet groups [a]

Dietary intake groups	HMHS eaters (n = 60)	HMLS eaters (n = 64)	LMHS eaters (n = 60)	LMLS eaters (n = 86)	*P*-values [c]
Meat	160.96 ± 59.88	205.58 ± 63.03	66.58 ± 21.42	40.31 ± 26.36	<0.001
Red meat	104.23 ± 52.14	141.93 ± 59.61	35.31 ± 18.73	14.27 ± 8.41	<0.001
Poultry	56.73 ± 38.21	63.65 ± 37.36	31.26 ± 16.59	26.04 ± 21.96	<0.001
Seafood	180.24 ± 45.39	78.51 ± 25.65	223.32 ± 59.96	34.38 ± 18.37	<0.001
Fish	147.30 ± 41.43	60.20 ± 24.53	198.30 ± 62.14	23.43 ± 13.81	<0.001
Shellfish	32.94 ± 23.29	18.31 ± 12.35	25.02 ± 15.92	10.95 ± 8.46	<0.001
Vegetables	249.49 ± 104.51	274.11 ± 103.86	261.32 ± 164.46	250.99 ± 187.03	0.763
Fruits [b]	285.17 ± 177.90	315.63 ± 212.91	411.10 ± 266.67	351.33 ± 216.22	0.013
Grains					
Whole grains [b]	4.74 ± 9.73	6.59 ± 11.20	6.72 ± 9.90	12.62 ± 18.88	0.003
Refined grains	383.68 ± 106.40	386.39 ± 124.93	390.48 ± 116.74	382.95 ± 114.40	0.982
Other					
Soy products [b]	12.05 ± 9.02	13.91 ± 10.94	16.65 ± 16.49	21.74 ± 24.50	0.005
Dairy	102.65 ± 161.16	90.75 ± 132.06	100.60 ± 136.56	145.92 ± 175.90	0.120

Eggs	32.03 ± 14.72	27.87 ± 18.08	30.08 ± 23.13	25.76 ± 18.49	0.222
Nuts and seeds	3.39 ± 4.07	3.04 ± 3.45	4.57 ± 5.19	4.50 ± 6.27	0.188
Total energy intake (kcal)	2125.30 ± 574.65	2058.24 ± 555.32	1896.30 ± 525.76	1970.50 ± 534.17	0.104

[a] HMHS, high meat and high seafood; HMLS, high meat and low seafood; LMHS, low meat and high seafood; LMLS, low meat and low seafood.

[b] *P*-values of 0.006 (fruits), 0.497 (whole grains), and 0.131 (soy products) were obtained when compared among HMHS, HMLS, and LMHS eaters.

[c] *P*-values were calculated by one-way ANOVA

Table S3. Optimized MRM conditions for determination of 16 amino acids

Compounds	R. T. (min)	Transition ion (m/z)	Fragmentor voltage (V)	CE (eV)
Phenylalanine	8.53	166.1 -> 120.1	90	14
Tryptophan	8.58	205.1 -> 146.0	70	18
Leucine	8.97	132.1 -> 86.1	90	10
Iso-leucine	9.61	132.1 -> 86.1	90	10
Methionine	10.44	150.1 -> 104.0	90	10
Tyrosine	11.52	182.1 -> 136.0	90	16
Valine	11.59	118.1 -> 72.1	90	10
Proline	11.61	116.1 -> 70.0	90	14
Hydroxyproline	12.59	132.1 -> 68.0	100	16
Alanine	12.67	90.1 -> 44.0	70	10
Threonine	13.37	120.1 -> 74.0	90	20
Glycine	13.77	76.0 -> 30.0	70	6
Glutamine	14.22	147.1 -> 84.0	70	16
Serine	14.26	106.1 -> 60.0	90	8
Asparagine	14.38	133.1 -> 74.0	70	14
Lysine	15.42	147.1 -> 84.0	90	14

Table S4. Optimized MRM conditions for determination of 19 fatty acid methyl esters

Fatty acid methyl esters	R. T. (min)	Transition ion (m/z)	CE (eV)
Pentadecylic acid methyl ester (C15:0)	11.00	257 -> 57	10
Palmitic acid methyl ester (C16:0)	11.80	271 -> 103	20
Palmitoleic acid methyl ester (C16:1)	12.89	269 -> 219	12
Margaric acid methyl ester (C17:0)	12.66	285 -> 103	10
cis-10-Heptadecenoic Acid methyl ester (C17:1)	12.99	283 -> 233	10
Stearic acid methyl ester (C18:0)	13.62	299 -> 103	20
Elaidic acid methyl ester (C18:1n9t)	13.78	297 -> 247	10
Oleic acid methyl ester (C18:1n9c)	13.91	297 -> 247	10
Linoleic acid methyl ester (C18:2n6c)	14.44	293 -> 95	15
γ-Linolenic acid methyl ester (C18:3n6)	14.78	293 -> 95	15
α-Linolenic acid methyl ester (C18:3n3)	15.12	293 -> 95	15
Arachidic acid methyl ester (C20:0)	15.70	327 -> 57	10
cis-11-Eicosenoic Acid methyl ester (C20:1n9)	16.02	325 -> 275	10
cis-11,14-Eicosadienoic Acid methyl ester (C20:2n6)	16.62	321 -> 81	10
cis-8,11,14-Eicosadienoic Acid methyl ester (C20:3n6)	16.99	321 -> 81	10
cis-11,14,17-Eicosatrienoic Acid methyl ester (C20:3n3)	17.37	321 -> 81	10
AA methyl ester (C20:4n6)	17.22	319 -> 95	10
EPA methyl ester (C20:5n3)	18.02	317 -> 161	18
DHA methyl ester (C22:6n3)	21.25	343 -> 95	20

Table S5. AUC values of 42 metabolites by comparing the top and the bottom quintiles of meat and seafood consumption

Metabolites	AUC (95% CI)
**Untargeted analysis**	
AA-content glycerophospholipids	
PC (36:4)	0.69 (0.59, 0.79)
PC (38:4)	0.68 (0.58, 0.78)
PC (p36:4)	0.75 (0.66, 0.85)
PC (o36:4)	0.71 (0.61, 0.81)
PC (o38:5)	0.70 (0.60, 0.80)
PE (p36:4)	0.78 (0.69, 0.87)
PE (p38:4)	0.73 (0.63, 0.83)
PE (p38:5)	0.73 (0.64, 0.83)
EPA-content glycerophospholipids	
PC (36:5)	0.84 (0.76, 0.91)
PE (p36:5)	0.84 (0.76, 0.91)
DHA-content glycerophospholipids	
LPC (22:6)	0.80 (0.71, 0.88)
PC (38:6)	0.85 (0.78, 0.92)
PC (40:6)	0.85 (0.78, 0.92)
PC (40:7)	0.67 (0.57, 0.78)
PC (o38:6)	0.82 (0.74, 0.89)
PC (p40:6)	0.80 (0.71, 0.88)

LPE (22:6)	0.74 (0.65, 0.84)
PE (38:6)	0.72 (0.63, 0.82)
PE (40:6)	0.76 (0.67, 0.85)
PE (40:9)	0.72 (0.62, 0.81)
PE (p38:6)	0.84 (0.77, 0.92)
non-AA/EPA/DHA-content glycerophospholipids	
PC (34:2)	0.66 (0.56, 0.76)
PC (36:2)	0.65 (0.55, 0.75)
PC (36:4)	0.67 (0.57, 0.77)
PC (o34:1)	0.69 (0.59, 0.79)
LPE (18:1)	0.65 (0.54, 0.75)
LPE (18:2)	0.74 (0.65, 0.83)
PE (36:3)	0.73 (0.63, 0.82)
PI (34:2)	0.74 (0.65, 0.84)
PI (36:2)	0.67 (0.57, 0.77)
PI (38:3)	0.71 (0.61, 0.81)
Other	
D-Glucose	0.71 (0.61, 0.81)
CMPF	0.80 (0.71, 0.88)
**Targeted analysis**	
Amino acids	
Glycine	0.64 (0.54, 0.75)
Hydroxyproline	0.80 (0.71, 0.88)

Lysine	0.67 (0.57, 0.77)
Threonine	0.59 (0.48, 0.70)
Tryptophan	0.72 (0.63, 0.82)
Valine	0.67 (0.57, 0.77)
Total fatty acids	
AA	0.71 (0.62, 0.81)
EPA	0.82 (0.74, 0.90)
DHA	0.85 (0.78, 0.92)

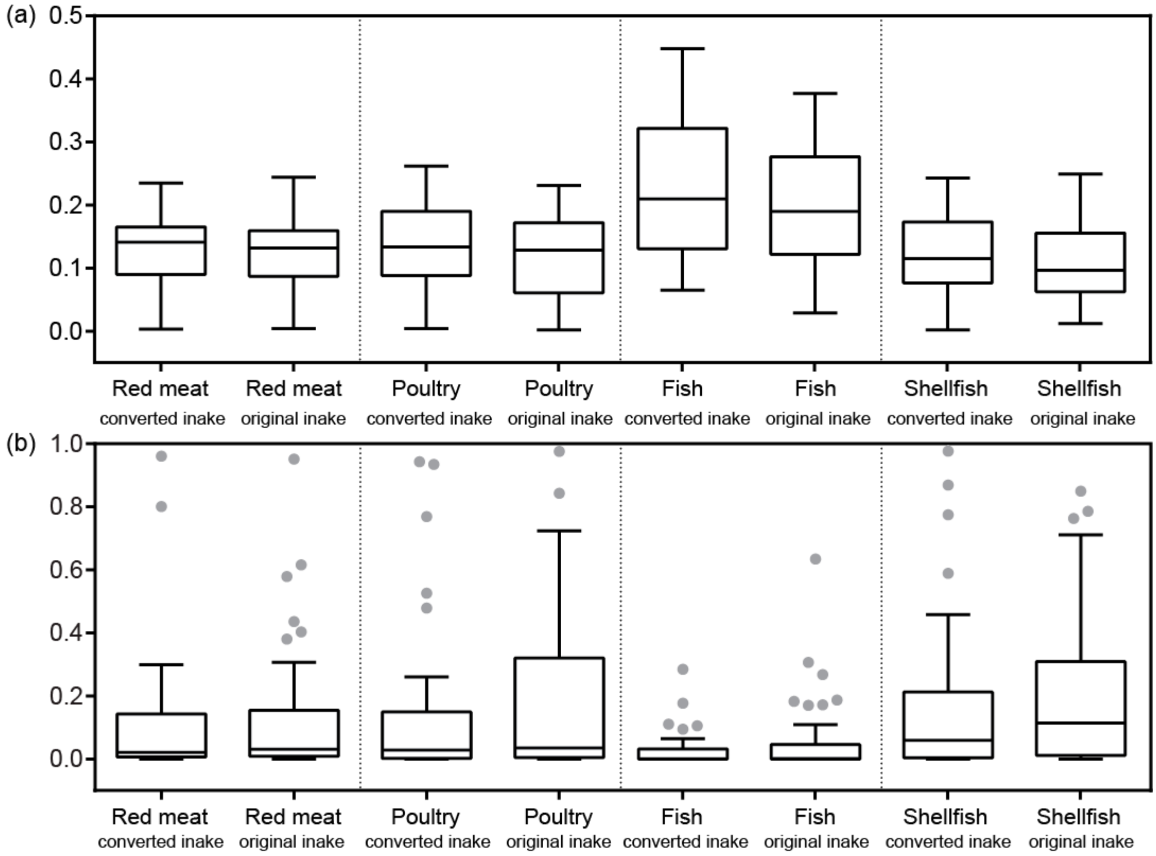

Figure S1. The association between food consumption and plasma metabolic profile: (a) Pearson correlation coefficient was calculated based on 42 differential metabolites, respectively; (b) the p values of correlation coefficient.

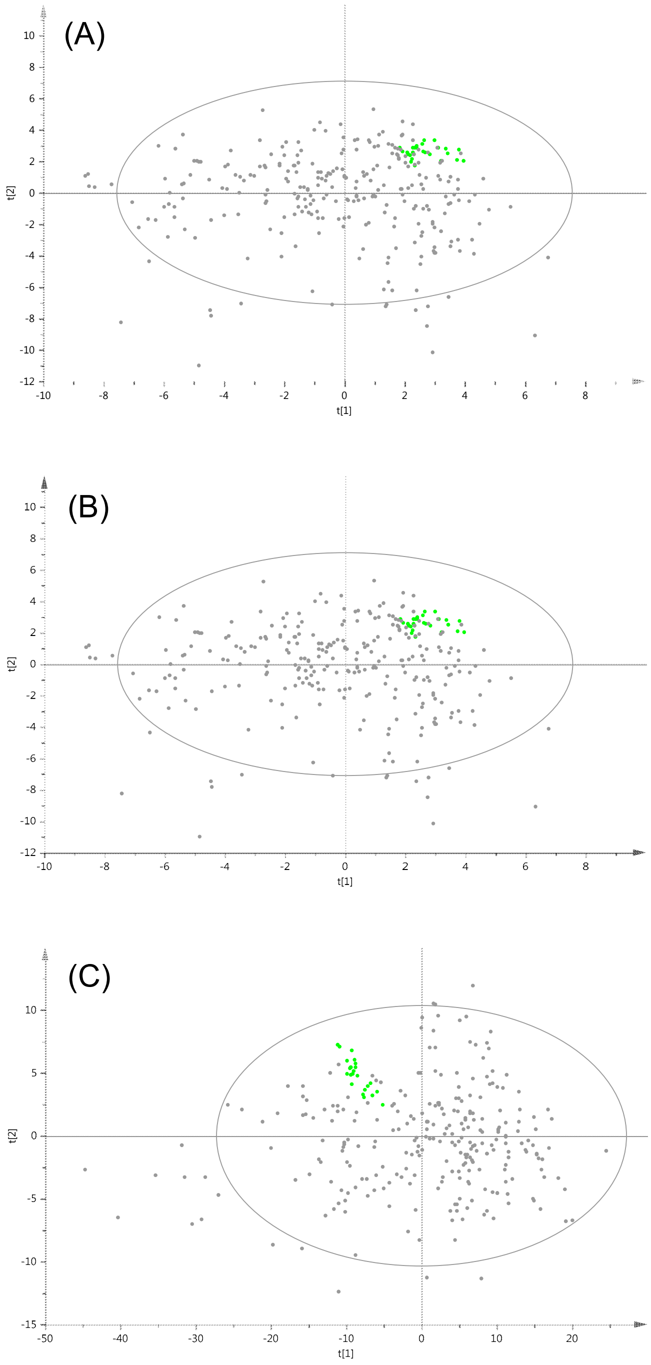

Figure S2. Principal component analysis (PCA) score scatter plots of all plasma (grey dots, n = 270) and QC (green dots, n = 27) samples. (A) LC-MS, positive ion mode, R2X (cum) = 0.344, Q2 (cum) = 0.134; (B) LC-MS, negative ion mode, R2X (cum) = 0.641, Q2 (cum) = 0.206; (C) GC-MS, R2X (cum) = 0.868, Q2 (cum) = 0.836.

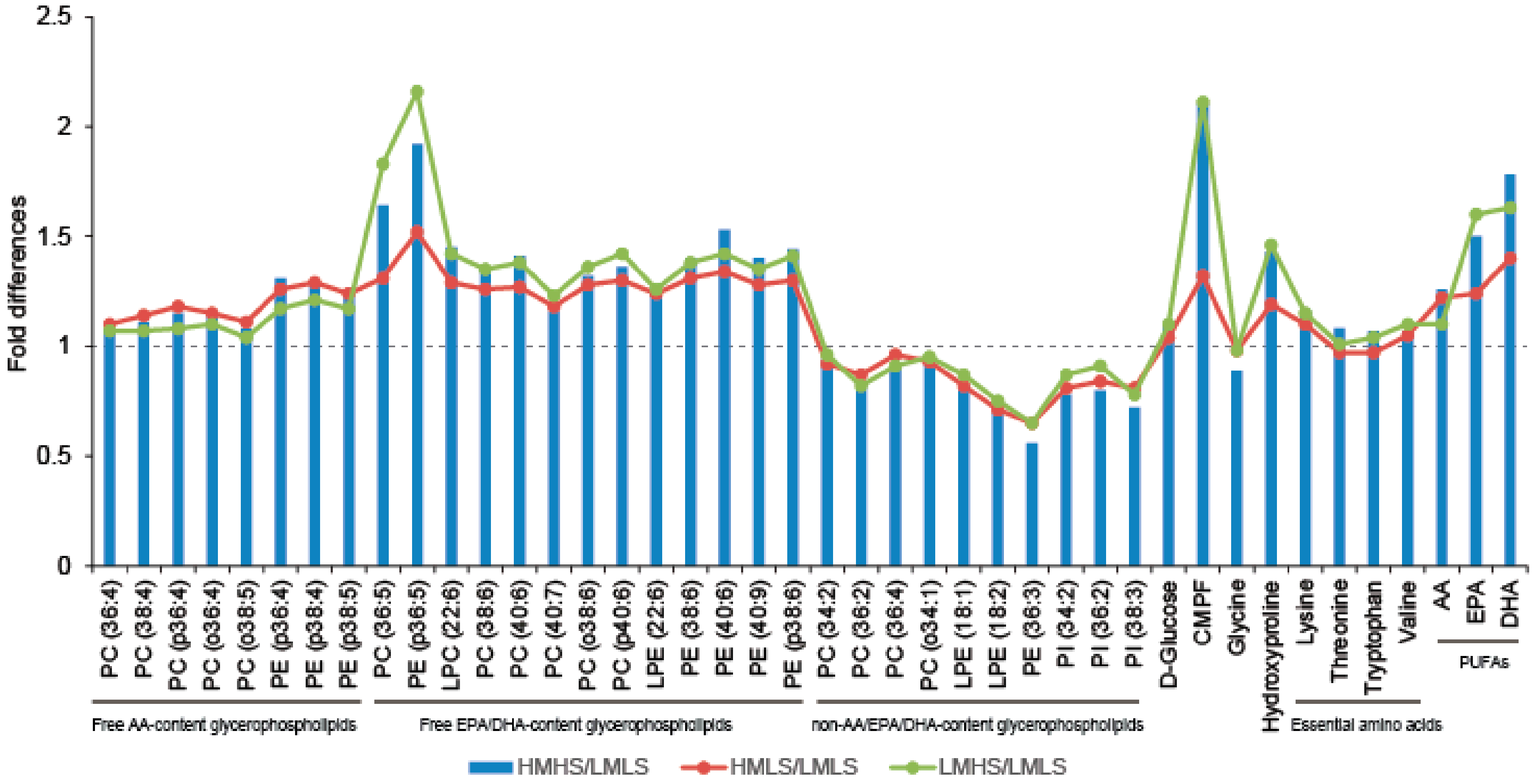

Figure S3. Fold differences of plasma metabolites in HMHS, HMLS and LMHS consumers by comparison with LMLS consumers. HMHS, high meat and high seafood; HMLS, high meat and low seafood; LMHS, low meat and high seafood, LMLS, low meat and low seafood.
